# The Editor's Letter-Box

**Published:** 1920-12-11

**Authors:** N. Howard Mummery

**Affiliations:** Lt.-Col., General Secretary. The Federation of Medical and Allied Societies, 5 Vere Street, W. 1


*To the Editor of* THE HOSPITAL.

SIR,—Will you allow me to comment on Lord Knutsford's letter in your last issue? His now famous, but erroneous, statement was made at a Conference of the Federation of Medical and Allied Societies on November 5, and those present were the representatives of most of the important organisations of those providing the medical, dental, pharmaceutical, nursing, midwifery, and allied services of the country. In addition to these the British Hospitals Association, individual hospitals, the Infirmary Medical Superintendents' Society, and Poor-law Infirmary Matrons' Association were represented, and several medical Members of Parliament attended on behalf of the Medical Committee of the House of Commons. Such an important, and probably unique, gathering can hardly be termed "a small meeting attended only by medical men," and one may well ask whether he has found members of that profession particularly inclined to believe in his prognostications.

As an uninvited, though honoured, guest Lord Knutsford was doubtless unaware of the nature of the Conference, and clearly has not read the full reports of it in the medical and lay press. Yours faithfully,

N. HOWARD MUMMERY, Lt.-Col., General Secretary.
The Federation of Medical and Allied Societies,
5 Vere Street, W. 1.
December 6, 1920.

